# Childhood Thyroid Radioiodine Exposure and Subsequent Infertility in the Intermountain Fallout Cohort

**DOI:** 10.1289/ehp.1104231

**Published:** 2012-10-25

**Authors:** Mary Bishop Stone, Joseph B. Stanford, Joseph L. Lyon, James A. VanDerslice, Stephen C. Alder

**Affiliations:** University of Utah Department of Family and Preventive Medicine, Salt Lake City, Utah, USA

**Keywords:** child, infertility, nuclear weapons, radioisotopes, sterility, thyroid disease

## Abstract

Background: Above-ground and underground nuclear weapon detonation at the Nevada Test Site (1951–1992) has resulted in radioiodine exposure for nearby populations. Although the long-term effect of environmental radioiodine exposure on thyroid disease has been well studied, little is known regarding the effect of childhood radioiodine exposure on subsequent fertility.

Objectives: We investigated early childhood thyroid radiation exposure from nuclear testing fallout (supplied predominantly by radioactive isotopes of iodine) and self-reported lifetime incidence of male or female infertility or sterility.

Methods: Participants were members of the 1965 Intermountain Fallout Cohort, schoolchildren at the time of exposure who were reexamined during two subsequent study phases to collect dietary and reproductive histories. Thyroid radiation exposure was calculated via an updated dosimetry model. We used multivariable logistic regression with robust sandwich estimators to estimate odds ratios for infertility, adjusted for potential confounders and (in separate models) for a medically confirmed history of thyroid disease.

Results: Of 1,389 participants with dosimetry and known fertility history, 274 were classified as infertile, including 30 classified as sterile. Childhood thyroid radiation dose was possibly associated with infertility [adjusted odds ratio (AOR) = 1.17; 95% CI: 0.82, 1.67 and AOR = 1.35; 95% CI: 0.96, 1.90 for the middle and upper tertiles vs. the first tertile of exposure, respectively]. The odds ratios were attenuated (AOR = 1.08; 95% CI: 0.75, 1.55 and AOR = 1.29; 95% CI: 0.91, 1.83 for the middle and upper tertiles, respectively) after adjusting for thyroid disease. There was no association of childhood radiation dose and sterility.

Conclusion: Our findings suggest that childhood radioiodine exposure from nuclear testing may be related to subsequent adult infertility. Further research is required to confirm this.

Many health outcomes may be affected by fallout from the detonation of nuclear weapons. Although the effects of gamma radiation have been examined extensively (Dropkin 2007; Otake et al. 1991), release of radioisotopes into the environment has received less attention. Among the isotopes released are large quantities of radioactive iodine. Human exposure occurs principally through the food chain, largely by milk consumption, but also by direct inhalation and skin contact. Exposure to radioiodine also occurs during medical ablative therapy for thyroid disease and subsequent outcomes have been studied (de Vathaire et al. 1997; Franklyn et al. 2005).

Environmental radioiodine exposure in the United States to date has resulted primarily from nuclear weapons detonated above ground at the Nevada Test Site from 1951 through 1958. Underground testing that began after 1958 and ended in 1992 also released radioiodine into the atmosphere by accidental venting. The National Cancer Institute (NCI) conducted a study of this exposure and demonstrated that the populations of 40 states received appreciable radioiodine exposure from 86 detonations (NCI 1997). The estimated total radioactivity from weapons testing at the Nevada Test Site was 5,600 petabecquerel (PBq). In comparison, the estimated releases from other sites such as the Hanford nuclear reprocessing facility (33 PBq) and the Chernobyl nuclear power station (1,700 PBq) were much lower, but occurred in a more concentrated time frame (Apostoaei and Miller 2004). In this article, we focus on the effects of the radioiodine component of the releases, because it is the primary and overwhelming source of radiation exposure to the thyroid.

The principal health concern of exposure to radioiodine has been thyroid disease because the thyroid gland concentrates iodine. Most but not all studies suggest an association between exposure to radioiodine and thyroid cancer (Cardis et al. 2005; Davis et al. 2004a, 2004b, 2006; Gilbert et al. 1998; Kerber et al. 1993; Lyon et al. 2006). In addition, some evidence points to an association with benign thyroid neoplasms and autoimmune thyroiditis (Eheman et al. 2003; Lyon et al. 2006), which is the most common form of thyroid disease affecting about 20% of the adult population of the United States (Lyon et al. 2006).

Possible outcomes of exposure to therapeutic radioiodine (e.g., to treat thyroid cancer), including subsequent fertility, have been studied in medical case series. Most of these studies were of adolescents or adults, and none estimated associations by individual doses. None of the studies found any association between exposure and fertility outcomes a year or later after treatment (Sawka et al. 2008a, 2008b). In populations exposed to the Chernobyl fallout, which included radioiodine, birth rates in nearby countries decreased 9 months after the fallout reached each country, suggested by researchers to be a result of a decrease in planning of pregnancy in the population during this time rather than necessarily an effect of the exposure (Auvinen et al. 2001).

We hypothesize that radioiodine exposure could affect subsequent fertility years beyond the time of exposure, either indirectly by affecting thyroid function, directly through effects on the gonads, or both. In addition to the thyroid gland, all organs including the male and female gonads are affected by environmental exposure to radioiodine (Ng et al. 1990; Whicker et al. 1996). Effects of radioiodine on thyroid disease have been found up to 40 years after the original exposure (Kerber et al. 1993). Thus, it is plausible that radioiodine exposure at an early age of development in childhood or *in utero* would have lasting effects on adult fertility. Here we report the first investigation of early childhood thyroid radiation exposure from environmental radioiodine and subsequent lifetime incidence of infertility or sterility, using dosimetry to estimate individual exposures.

## Materials and Methods

*The Intermountain Fallout Cohort*. In 1965, to address the possibility of a relationship between thyroid cancer and exposure to radioiodine, the federal government through the Bureau of Radiological Health identified a cohort of 4,818 schoolchildren in grades 6–12 who had varying exposure to radioiodine from nuclear weapons tested above ground at the Nevada Test Site (phase I cohort). The children were residents of Washington County, Utah, and Graham County, Arizona, in 1965. In the initial analyses, the children living in Washington County were considered exposed at the time and those in Graham County were considered unexposed. In 1966 schoolchildren in Lincoln County, Nevada, were added to the cohort and were considered exposed (Simon et al. 1990). The cohort was located and reexamined in the mid-1980s (phase II). Information about residence and milk and vegetable consumption was obtained from the parents of the cohort members to calculate individual thyroid radioiodine doses for 3,545 members of the cohort. In 1993, investigators reported an association between exposure to radioiodine and thyroid neoplasms (Kerber et al. 1993).

*Participants*. The participants of this study were members of the original cohort established in 1965 (phase I, *n* = 4,818), as well as of the follow-up to this cohort with medical thyroid examinations in 1985–1986 (phase II, *n* = 4,285), and a subgroup with further updated dosimetry calculations (phase IIR, *n* = 2,497). We again located and examined the cohort beginning in 2003 (phase III), and, as part of the examinations, we obtained new informed consent and administered a medical history questionnaire, which included, for the first time, a section on reproductive history. The questionnaire included specific questions about all sexual partnerships lasting ≥ 6 months, and based on these questions we determined that some members of the original cohort were married or were sexual partners with each other.

*Data collection*. Once contact had been established, we scheduled examination appointments for participants at a clinic site near the University of Utah. Participants were asked to complete the medical history questionnaire before they came to their appointment. We asked them to consult their own records as they completed the questionnaire. The clinic visit included a physical examination and ultrasound examination of the thyroid gland. If the participants had not completed the questionnaire at the time of their appointment, we asked them to complete it after the examination and before leaving the clinic. Most people completed the questionnaire before arriving at their appointment.

The completed questionnaires were reviewed by a study staff member. If any of the information in the questionnaire was incomplete, inconsistent, or not understandable and it was not corrected at the time of the clinic visit, the participant was contacted by phone to clarify the information. The questionnaires were then double entered into a Microsoft Access database (Microsoft Corporation, Redmond, WA) by two staff members, and the two data streams were compared by a third staff member.

*Dosimetry*. Many of the data used in our models came from work done by many over the previous decades (Anspaugh et al. 2002; Beck and Krey 1983; Beck et al. 2006; Hicks 1982; Hoffman 1977, 1978; Mallick et al. 2002; Miller et al. 1978; Ng et al. 1990; O’Neill et al. 1981; Rosenstein 1982; Simon et al. 1990, 2006a; Thompson 1990; Whicker and Kirchner 1987). The dosimetry model included calculations for inhalation and external pathways, and included all isotopes of radioiodine, including I-129, I-131, and I-133, as well as external gamma radiation. Because the vast majority of the radiation exposure was contributed by radioioidine, with I-131 being the predominant component (Lyon et al. 2006), we refer to the exposure as radioiodine.

The original dosimetry calculations and mathematical models done in phase II have been reported in detail (Simon et al. 1990; Till et al. 1995). In brief, radioactive debris deposition was obtained from 97 gummed film stations that had been placed in various locations around the country at the time of the above ground testing (Beck and Krey 1983; Beck et al. 2006). The amount of debris that would remain on the vegetation once it was deposited was estimated (Simon et al. 1990). Cows’ milk production and distribution were estimated for the western United States (Whicker and Kirchner 1987) and for the rest of the United States (NCI 1997). Milk production in the three counties of the study was estimated by interviewing local dairy farmers about their years of production, herd sizes, feeding practices, and hay production.

Of the 4,285 cohort members located in phase II, information necessary for individual dosimetry was obtained from the parents of 3,545 of the subjects. Mothers completed 90% of the interviews. Estimates of the direct consumption of contaminated vegetation were made by asking parents of cohort members about their home garden production of green leafy vegetables. The parents provided such information as how often their children ate the vegetables and during which months these vegetables were grown.

To estimate how much fresh milk the members of the cohort consumed from birth, the parents were asked to estimate daily milk consumption for specific age groups. If the child had been *in utero* during the weapons testing, the mother’s fresh milk consumption was also estimated. The parents were also asked to recall the source of the fresh milk, such as a backyard cow or goat, neighbor, local dairy, or grocery store, by name and location. “Backyard” cows or goats were those kept by a family to supply milk to the family and perhaps a neighbor.

In phase IIR, investigators revised and updated the dosimetry model to correct previous calculation errors discovered in phase II and update some parameters of the model. New doses were calculated for individual cohort members. To be certain that the complex mathematical models were programmed according to the specifications of the dosimetrists, the models were programmed independently in SAS version 9.1.2 (SAS Institute Inc., Cary, NC) and in Analytica 3.0 Enterprise (Lumina Decision Systems, Los Gatos, CA), and intermediate results were compared. When discrepancies were identified, the programming variation was identified and traced to error or to misunderstanding of the dosimetry specification, and corrected. The changes to the model for phase IIR have been reported previously (Simon et al. 2006a, 2006b, 2006c), as has the subsequent reanalysis of thyroid disease outcomes for the 2,497 cohort members (Lyon et al. 2006).

*Infertility*. We asked women “Have you ever tried to become pregnant for a year or longer?” Men were asked “Have you ever tried for a year or longer to father a pregnancy?” Both women and men who answered yes were then asked “How old were you when this first occurred?” We considered infertility to exist if the respondent had answered yes, and that this happened at < 40 years of age. This definition of infertility does not preclude a pregnancy occurring either before or after the episode(s) of infertility (Larsen 2005).

*Sterility*. Sterility is defined as the permanent inability to conceive (Gnoth et al. 2005). Technically, this requires identification of a condition precluding the ability to conceive. However, sterility is inferred in epidemiological or demographic studies at the end of the reproductive life in which there has been opportunity to conceive. All of the women in this study were at or near the end of their reproductive lives. We therefore defined sterility as the absence of any pregnancy, despite having tried to become pregnant (or cause a pregnancy) for a year or longer before 40 years of age. Thus, we considered those who were classified as sterile to be a subset of those who were classified as infertile.

*Thyroid disease*. Thyroiditis, hyperthyroidism, and hypothyroidism have been associated with female and male fertility (Abalovich et al. 2007; Krassas and Pontikides 2004), and are also possible outcomes of radioiodine exposure (Eheman at al. 2003). We therefore included thyroid disease in some of our models to investigate its potential role as a mediating variable. For our analyses, we combined all these types of thyroid disease into a single dichotomous variable. All thyroid disease was medically confirmed by expert review of medical records (Lyon et al. 2006).

*Statistical methods*. For a descriptive overview, we graphed the incidence of infertility (ever vs. never infertile, as defined above) by radioiodine doses to the thyroid in 20 percentile groupings (i.e., ordered dose categories with 5% of the participants in each category). We modeled the relationship between radioiodine dose to the thyroid in Grays and infertility using standard multivariable logistic regression, with exposure modeled as a continuous variable or categorized according to tertiles or quartiles of the exposure distribution. Covariates adjusted for in the model include sex, total number of years in marriage or other sexual partnership, history of smoking dichotomized to > 1 pack-year history versus ≤ 1 pack-year by 35 years of age, history of any alcohol consumption versus none by 35 years of age, age at pregnancy or pregnancy attempt, body mass index based on self-reported height and weight about the time of attempting pregnancy, education, and history of radiation exposure at work. Each of these covariates has a known or likely association with infertility. Since all cohort members were > 40 years of age at the time of the phase III assessment, we did not adjust for current age in our regression models.

To account for the potential clustering among couples in the cohort, we used a sandwich estimator to derive robust confidence intervals (Arminger 1995; Liu 1998). Logistic regression modeling was performed using Stata version 12.1 (StataCorp, College Station, TX).

*IRB approval*. The phase III study was approved by the University of Utah Institutional Review Board and the U.S. Centers for Disease Control and Prevention Institutional Review Board.

## Results

The original cohort had 4,818 members. During phase III, we located 4,648 (96.5%) of the 4,818 cohort members as of August 2005, and started contacting them to schedule study visits (questionnaires and examinations). When the phase III visits ended because funding ended earlier than anticipated, we had contacted 2,412 persons (51.9% of those who were located), of whom 5.5% refused participation. We also found that 46 cohort members were deceased. At the time study visits ended, we had received questionnaires from 1,596 participants (33.1% of the original cohort and 66.2% of those who had been contacted during phase III).

**Figure 1:**
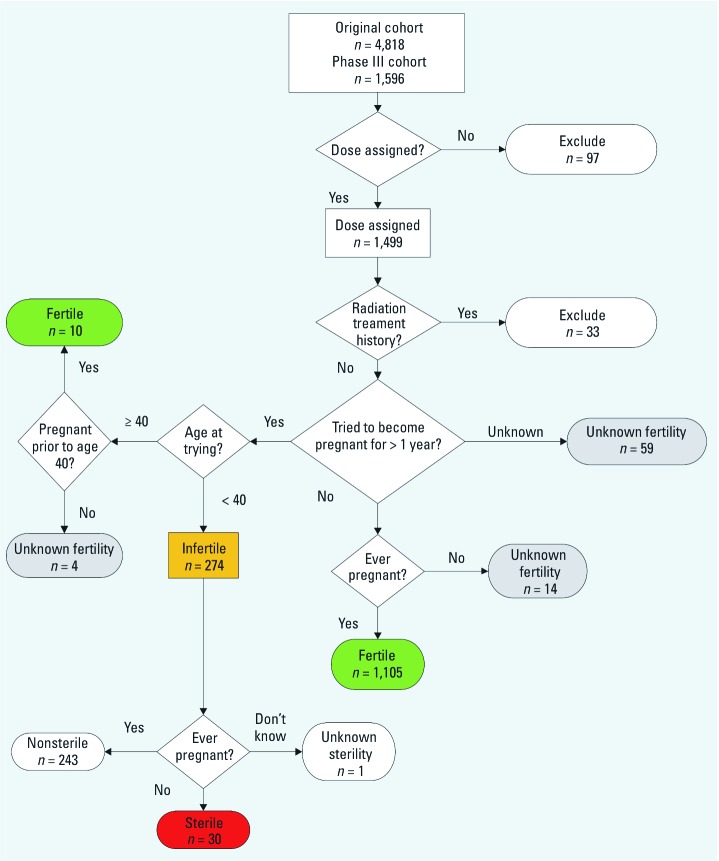
Criteria used to determine eligibility and fertility outcomes among cohort members.

Of the 1,596 participants with phase III questionnaire information, we excluded 97 participants who did not have calculated individual radioiodine doses from phase II, and 33 participants who had prior radiation treatment, leaving 1,466 participants who were classified as fertile (*n* = 1,115 fertile) or infertile (*n* = 274), and 77 participants of unknown fertility status (59 because they didn’t know whether they had ever tried to get pregnant for a year or more, 14 because they had never tried to get pregnant for a year or more and had never been pregnant, and 4 because they had never been pregnant and first attempted to become pregnant when ≥ 40 years of age) (Figure 1). Of the 274 infertile participants, 30 were classified as sterile.

Phase I participants (*n* = 4,818) were 50.2% male, whereas phase III participants included in this analysis (*n* = 1,466) were 45.2% male (Table 1). The birth year distribution of phase I participants (range, 1944–1957; median, 1951) was very similar to that for phase III participants (range, 1945–1955; median, 1952). In addition, the radioiodine dose distribution was similar between phase III participants (*n* = 1,466) and phase IIR participants (*n* = 2,497): mean ± SD, 117.1 ± 172.3 mGy; median, 50.8 mGy; and 120 ± 167 mGy; median, 55.0 mGy, respectively. As of phase III, 10.0% of the original 4,818 cohort members had married or were partners with each other. In the analysis subcohort reported here, 17.2% were married or were partners with each other.

**Table 1:** Characteristics of phase III cohort members included in analysis (*n* = 1,466).

Characteristic	Mean ± SE or n (%)
Age at the time of phase III assessment (years)		52.0 ± 2.3
Sex		
Female		804 (54.8)
Male		662 (45.2)
Race/ethnicity		
Non-Hispanic white		1353 (92.3)
Hispanic		112 (7.6)
Other		1 (0.1)
Radioiodine dose to thyroid (mGy)		117.1 ± 172.3
0.1–17.5 mGy		489 (33.3)
17.6–101.0 mGy		489 (33.3)
101.0–1245.5 mGy		488 (33.3)
Time in sexual partnership (years)a		27.6 ± 7.1
Age at pregnancy attempt (years)a,b		22.7 ± 4.1
Cigarette smoking (> 1 pack-year through age 35 years)a		
Yes		299 (20.5)
No		1,162 (79.5)
Alcohol use (any use through age 35 years)a		
Yes		456 (31.2)
No		1,006 (68.8)
Body mass index about the time of pregnancy attempta		
Underweight (< 18.5)		255 (17.9)
Normal (18.5–24.9)		842 (59.2)
Overweight (> 24.9)		326 (22.9)
Education		
High school graduate or less		301 (20.5)
Some college or technical school		664 (45.3)
College graduate		303 (20.7)
Postgraduate		198 (13.5)
History of working with or around radiationa		
Yes		170 (11.6)
No		1,294 (88.4)
Thyroid disease (medically confirmed thyroiditis, hyperthyroidism, or hypothyroidism)b		
Yes		103 (7.4)
No		1,296 (92.6)
aSome participants had missing data for the following variables: time in sexual relationship(s) (n = 44), age at pregnancy or pregnancy attempt (n = 37), smoking (n = 5), alcohol (n = 4), body mass index (n = 23), history of radiation (n = 2), thyroid disease (n = 67). bDefined as either age when tried for 1 year to get pregnant, or the age when first got pregnant, or the age when married (in priority order).		

**Figure 2:**
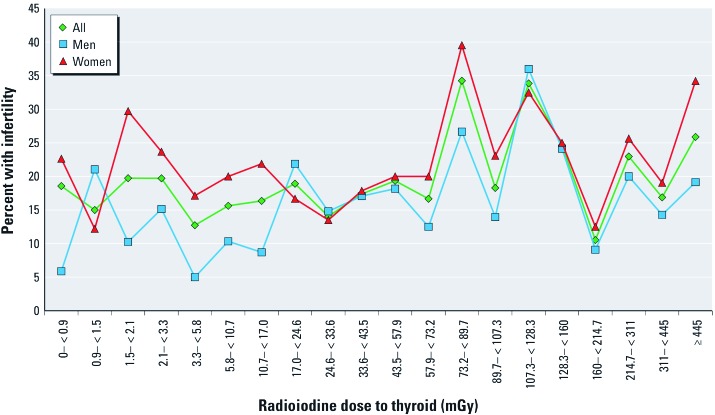
Unadjusted incidence of infertility by sex and by 20 percentiles (5% increments) of childhood radioiodine dose.

For the analyses of fertility outcomes, we excluded the 77 participants with unknown fertility status, leaving 1,389 persons available. The unadjusted distribution of infertility by radioiodine dose is displayed in Figure 2. We conducted subsequent analyses with the dose divided into tertiles or quartiles, as well as with dose as a continuous variable.

The unadjusted odds ratios for infertility for the second and third dose tertiles compared with the first were 1.22 (95% CI: 0.88, 1.70) and 1.33 (95% CI: 0.95, 1.84), respectively. Corresponding adjusted odds ratios (AOR) from models including all covariates except thyroid disease were 1.17 (95% CI: 0.82, 1.67) and 1.35 (95% CI: 0.96, 1.90) When medically confirmed thyroid disease was added to the model, the corresponding AOR attenuated slightly: 1.08 (95% CI: 0.75, 1.55) and 1.29 (95% CI: 0.91, 1.83), respectively. In the latter model, thyroid disease itself was a significant risk factor for infertility: AOR = 1.78 (95% CI: 1.13, 2.82) (Table 2). We also investigated the association of thyroid disease with infertility in an adjusted model that did not include radioiodine dose but was otherwise the same as that shown in Table 2. This yielded a similar estimate for thyroid disease: AOR = 1.84 (95% CI: 1.16, 2.92).

**Table 2:** AORs (95% CIs) for self-reported lifetime infertility, without or with thyroid disease as a covariate.

Characteristic	Without thyroid disease	With thyroid disease
Sex				
Female versus male		1.64 (1.20,2.24)		1.52 (1.10,2.10)
Radioiodine dose to thyroid (referent tertile: 0.1–17.5 mGy)				
17.6–101.0 mGy		1.17 (0.82,1.67)		1.08 (0.75,1.55)
101.0–1245.5 mGy		1.35 (0.96,1.90)		1.29 (0.91,1.83)
Time in sexual partnership (years)		1.01 (0.99,1.04)		1.01 (0.99,1.04)
Cigarette smoking (dichotomous)		1.25 (0.81,1.92)		1.23 (0.79,1.91)
Alcohol use (dichotomous)		1.02 (0.72,1.46)		1.01 (0.70,1.46)
Age at pregnancy attempt (years)		1.06 (1.02,1.11)		1.06 (1.01,1.11)
Body mass index about the time of pregnancy attempt (referent 18.5–24.9)				
Underweight (< 18.5)		0.79 (0.55,1.13)		0.79 (0.55,1.15)
Overweight (> 24.9)		0.66 (0.41,1.05)		0.67 (0.41,1.08)
Education (referent: high school graduate or less)				
Some college/technical school		1.62 (1.08,2.43)		1.64 (1.08,2.48)
College graduate		1.86 (1.17,2.96)		1.83 (1.14,2.94)
Postgraduate		1.10 (0.64,1.91)		1.14 (0.66,1.98)
History of working with or around radiation		1.23 (0.81,1.85)		1.27 (0.84,1.92)
Thyroid disease		—		1.78 (1.13,2.82)

In models with the radioiodine dose divided into quartiles, the unadjusted odds ratios for the second, third, and fourth quartiles (in order of increasing dose) were 1.04 (95% CI: 0.70, 1.54), 1.64 (95% CI: 1.13, 2.38), and 1.21 (95% CI: 0.82, 1.78), respectively. The corresponding AORs were 1.16 (95% CI: 0.74, 1.69), 1.62 (95% CI: 1.09, 2.41), and 1.27 (95% CI: 0.85, 1.90), respectively. In the adjusted model with thyroid radioiodine dose as a continuous variable, the AOR for dose (milliGrays) was 1.000 (95% CI: 1.000, 1.001).

We found no association between radioiodine dose and sterility (AOR = 1.02; 95% CI: 0.35, 2.94 and AOR = 1.11; 95% CI: 0.42, 2.87 for the middle and upper tertiles vs. the first tertile, respectively, adjusted for all covariates except thyroid disease).

## Discussion

In this exploratory study, we found an association between at least one episode of infertility in adulthood and environmental thyroid radioiodine exposure in childhood. To our knowledge, this is the first report of such an association.

This cohort is unique in its characterization of both childhood exposure to environmental radioiodine (for those completing phase II) and adult fertility outcomes (for those completing phase III). In a cohort study of the radioactive fallout of Hiroshima and Nagasaki, children exposed *in utero* who grew up were less likely to marry than nonexposed contemporaries, although those who did marry did not have significantly fewer children (Blot et al. 1975).

We had no biological or epidemiologic data sufficient to guide selection of dose categories for fertility outcomes. In previous work in this cohort, we found evidence for a nonlinear threshold effect regarding childhood thyroid radioiodine exposure and adult thyroid disease, using four dose categories (Lyon et al. 2006). However, we did not necessarily expect the dose–response relationship for fertility to be the same as for thyroid disease. We therefore approached the selection of dose categories *de novo*, using tertiles and quartiles of dose. In alternative analysis that assumed a linear dose response, we did not observe an association. Importantly, dose–response relationships between other health outcomes and thyroid radiation exposure have been reported to be nonlinear (Dropkin 2007; Stevens et al. 1990).

An association between infertility and thyroid and/or whole body radioiodine exposure is biologically plausible for both sexes, through several different mechanisms and pathways. The testis is known to be highly radiosensitive (Bonde et al. 1996). Medical exposure to radioiodine can result in transient failure of spermatogenesis and, at higher doses, possibly permanent azospermia (Hyer et al. 2002; Wichers et al. 2000). The ovaries are also sensitive to radioiodine, and medical exposure can lead to transient ovarian failure (Vini et al. 2002). Thus an exposure to radioiodine could result in infertility.

The gonads also have thyroid hormone receptors (Poppe et al. 2007). However, in adjusted models (Table 2), thyroid disease was nearly an independent risk factor for infertility. Thus, our results suggest a possible impact of childhood radioiodine exposure independently of known thyroid disease (hypothyroidism, hyperthyroidism, and thyroiditis). We did not have sufficient sample size to analyze different thyroid conditions separately.

Associations between covariates and infertility were somewhat consistent with previous research. Female sex was associated with infertility, consistent with infertility being somewhat more likely to be related to female than male causes (Evers 2002). Increasing age at which the participants first tried for a year or longer to become pregnant was significantly associated with infertility, as expected (Dunson et al. 2004). We did not find an association between smoking and infertility, but the prevalence of smoking was low in this cohort.

Follow-up of the cohort for dosimetry and reproductive outcomes was incomplete because of the termination of funding. This raises the question of whether there could be selection bias. However, follow-up was scheduled based on logistical considerations, without any regard to dose or outcome, examination clinics were distributed proportionally among population centers, and the dose distribution and demographic characteristics of the phase III cohort were very similar to the parent cohort.

The study has several limitations. The dosimetry model was based on inputs with multiple sources of measurement error including release estimates, data on environmental dispersion, dietary recall of usual milk consumption from mothers, and assumptions about bioavailability and biokinetics. However, we expect the error from these sources to be nondifferential with respect to outcome. We previously modeled the impact of the errors in the dosimetry model; in brief, accounting for uncertainties in each subject’s exposure will increase the magnitude of the AOR of disease for a given exposure, and will widen the CI around the odds ratio (Lyon et al. 2006; Mallick et al. 2002). The dosimetry model does not yield doses to the gonads or the uterus, which would also be relevant for these outcomes. However, we believe gonadal doses would be proportional to the thyroid doses.

In analyzing results by quartiles, we found a significant association of the third quartile with infertility, compared with the lowest quartile: unadjusted OR = 1.64 (95% CI: 1.13, 2.38); AOR = 1.62 (95% CI: 1.09, 2.41). However, we found a somewhat lower risk of infertility associated with the highest versus lowest quartile: AOR = 1.27 (95% CI: 0.85, 1.90) (Figure 2). The assessment of the potential impact of a full range of radioiodine exposure and infertility in this cohort is hampered by a relative paucity of participants with very high exposures, with wider dose ranges for the upper tertile (101.0–1245.5 mGy) or quartile (128.3–1245.5 mGy). A larger sample size would allow for more precision in addressing the association at higher doses. We regret that funding did not allow for us to complete the phase III follow-up for all the phase II cohort participants that we had located.

Episodes of infertility and sterility were retrospectively recalled, which can lead to bias; however, the questions we used have been used in many other retrospective studies of fertility have been shown to have good reliability (Joffe et al. 2005). A validation study comparing prospectively determined time to pregnancy and a later retrospective determination, with a median recall of 14 years, found 80% sensitivity and 95% specificity for infertility (time to pregnancy of ≥ 12 months) (Joffe et al. 1993). We do not know the partner’s fertility status (unless both were in the study), which may have resulted in some misclassification of fertility, but we expect this misclassification to be nondifferential with respect to the participant’s exposure; in other words, we do not expect that the likelihood of a partner to be fertile would be related to the participant’s exposure status. We also do not have detailed information about the time trying for pregnancy (beyond the 1 year of infertility), or about periods of contraceptive use, which would help confirm how much time each person had when infertility and sterility could potentially be detected. However, we excluded participants who never had an opportunity to test their fertility (Figure 1), so the ascertainment of lifetime incidence of at least one episode of infertility did not depend on information about contraceptive use.

This study has several strengths. We had individual dose calculations based on dietary intake and real-time ground fallout measures from 97 stations, in contrast with other studies, such as the studies of nuclear plant accidents (Davis et al. 2004a, 2004b). The dosimetry modeling has been reviewed and reported in the peer-reviewed literature (Mallick et al. 2002; Simon et al. 1990, 2006a, 2006b, 2006c; Till 1997; Till et al. 1995). Cohort members did not have access to their own dosimetry information through the study, and fertility outcomes were not the main focus of the health questionnaire, mitigating concerns about recall bias. The study had low refusal rates, and the sample is representative of the original phase I cohort and the populations of the three original counties (Rallison et al. 1990).

## Conclusions

In this exploratory analysis, we have found a possible association between childhood radioiodine exposure and subsequent infertility. If confirmed, this finding has potentially broad impact, since an estimated 55 million children were exposed in the United States (Hoffman et al. 2002). We recommend that this relationship be evaluated in the full cohort when funding allows, and in other populations who have been exposed to environmental contamination with radioiodine in order to corroborate or refute our findings.

## Correction

In the manuscript originally published online, the tables were numbered incorrectly in the text. The errors have been corrected here.
